# Radiomic and clinical nomogram for cognitive impairment prediction in Wilson’s disease

**DOI:** 10.3389/fneur.2023.1131968

**Published:** 2023-04-28

**Authors:** Liwei Tian, Ting Dong, Sheng Hu, Chenling Zhao, Guofang Yu, Huibing Hu, Wenming Yang

**Affiliations:** ^1^Graduate School, Anhui University of Traditional Chinese Medicine, Hefei, China; ^2^Department of Neurology, The First Affiliated Hospital of Anhui University of Traditional Chinese Medicine, Hefei, China; ^3^Key Laboratory of Xin’An Medicine, Ministry of Education, Hefei, Anhui, China; ^4^Centers for Biomedical Engineering, University of Science and Technology of China, Hefei, China; ^5^Qimen People's Hospital, Huangshan, Anhui, China

**Keywords:** Wilson’s disease, radiomics, cognitive impairment, machine learning, MRI

## Abstract

**Objective:**

To investigate potential biomarkers for the early detection of cognitive impairment in patients with Wilson’s disease (WD), we developed a computer-assisted radiomics model to distinguish between WD and WD cognitive impairment.

**Methods:**

Overall, 136 T1-weighted MR images were retrieved from the First Affiliated Hospital of Anhui University of Chinese Medicine, including 77 from patients with WD and 59 from patients with WD cognitive impairment. The images were divided into training and test groups at a ratio of 70:30. The radiomic features of each T1-weighted image were extracted using 3D Slicer software. R software was used to establish clinical and radiomic models based on clinical characteristics and radiomic features, respectively. The receiver operating characteristic profiles of the three models were evaluated to assess their diagnostic accuracy and reliability in distinguishing between WD and WD cognitive impairment. We combined relevant neuropsychological test scores of prospective memory to construct an integrated predictive model and visual nomogram to effectively assess the risk of cognitive decline in patients with WD.

**Results:**

The area under the curve values for distinguishing WD and WD cognitive impairment for the clinical, radiomic, and integrated models were 0.863, 0.922, and 0.935 respectively, indicative of excellent performance. The nomogram based on the integrated model successfully differentiated between WD and WD cognitive impairment.

**Conclusion:**

The nomogram developed in the current study may assist clinicians in the early identification of cognitive impairment in patients with WD. Early intervention following such identification may help improve long-term prognosis and quality of life of these patients.

## 1. Introduction

Wilson’s disease (WD) is a rare autosomal recessive disorder clinically characterized by abnormalities in copper metabolism (1, 2). If left untreated, WD initially presenting with liver disease may progress to a multisystem disease with neurological involvement (3). The predominant neurological symptoms of WD are extrapyramidal movement disorders, which may be accompanied by varying degrees of cognitive impairment. Although some studies have indicated that individuals with WD demonstrate widespread impairments in cognition, especially prospective memory (PM) impairments, few cases of WD cognitive impairment have been described in the literature (4). However, in clinical settings, the administration of neuropsychological assessments is susceptible to the influence of uncontrollable factors that may introduce bias into the data. Furthermore, the symptoms of WD cognitive impairment are easily masked, and clinical features may escape detection given interindividual variations in presentation. This increases the difficulty of establishing a clinical diagnosis and can lead to a lack of objectivity and consistency when making a diagnosis based on neuropsychological scales alone (5, 6).

We conducted a series of studies to investigate the issue of cognitive impairment in WD. Notably, these studies revealed that cognitive impairment was dominated by a disruption in PM, which refers to the ability to remember to do something in the future. Patients with WD appear to have greater difficulty with time-based PM (TBPM), which encompasses the memory to do something in the future after a certain period of time has passed, as opposed to event-based PM (EBPM), which references one’s memory to do something in the future after a specific event occurs. Consistent with this, white matter damage in patients with WD occurs mainly in subcortical white matter brain regions, with extensive damage to white matter trajectories in the limbic system as well as those involved in PM (7). Subsequent structural and functional imaging studies demonstrated that gray-matter volumes, including the hippocampus and basal ganglia, are significantly reduced in patients with WD cognitive impairment compared to neurotypical samples, and that global cognitive status may be indirectly influenced by the functional connectivity among visual association cortex, thalamus, and hippocampus (8, 9).

Advancements in MRI technology and imaging resolution have led to the widespread adoption of various MRI sequences for diagnosis and evaluation of neurodegenerative diseases in clinical practice (10–13). Recent medical imaging research has emphasized the value of radiomics analysis, especially in patients with neurological diseases (14). This relatively new technique can aid in extracting original image features that are relevant to the identification and quantification of different cognitive states (15, 16). Thus, radiomic characterization based on image features may aid in differentiating WD cognitive impairment from WD. However, this remains difficult due to insufficient models that incorporate clinically-meaningful, high-risk factors.

Based on the group’s past research, this study used radiomics as a research method to identify risk factors, independent predictors, and imaging markers of WD cognitive impairment through non-invasive, reproducible means as well as machine learning to build a diagnostic model that provides a strong theoretical basis for a clinical diagnosis of WD cognitive impairment. The predictive ability of this model can be used to assess whether radiomic features extracted from structural images can improve the accuracy of distinguishing between WD cognitive impairment and WD.

## 2. Materials and methods

### 2.1. Patient data

The images used in this study were obtained from the First Affiliated Hospital of the Anhui University of Chinese Medicine (AUCM). WD was diagnosed by experienced neurologists based on clinical characteristics, confirmed abnormalities in copper metabolism, and neuroimaging results. Patients with cognitive impairment, as assessed using a neuropsychological inventory, were classified into the WD cognitive impairment group. All participants were treated with penicillamine and zinc salts, in accordance with standard medical protocol. All eligible participants underwent brain MRI and provided written informed consent. In total, we analyzed 77 and 59 images from patients with WD and WD cognitive impairment, respectively. These images were then randomly distributed into training and test groups at a ratio of 70:30. Data collection for this study was approved by the AUCM Ethics Committee (number: 2019AH-08).

### 2.2. Neuropsychological evaluation

Two neuropsychologists independently administered neuropsychological tests to each participant, and the final scores were averaged for analysis. The Chinese version of Addenbrooke’s Cognitive Inventory-III (ACE-III-C) was used to assess cognitive dysfunction in the sample, which parses cognition into five domains: attention and orientation (out of 18 points), memory (out of 26 points), fluency (out of 14 points), language (out of 26 points), and visual–spatial functioning (out of 16 points). In addition to generating subscale scores, a total score is calculated out of 100 points, with higher scores indicating better cognitive function and a summary score <88 considered indicative of cognitive dysfunction (17, 18). As the ACE-III-C is an extension of the traditional Mini Mental State Exam (MMSE), administration of the ACE-III-C permits simultaneous calculation of a MMSE score out of 30 points; in this study, the MMSE score was used as an index of temporal and spatial abilities, short-term memory, and visual functioning (19, 20).

In addition to the ACE-III-C, EBPM, and TBPM tests were administered to assess the degree of impairment in PM, as previously described (21). In the EBPM test, participants were first provided with a task event and asked to include their contact information at the end of the test. Patients were required to identify the task event as tapping on the table during the test. Thirty cards were presented to the patients; of the 12 words on the cards, 10 belonged to one category, while the remainder belonged to another. When the experimenter presented the cards, the participant was asked to select two words belonging to different categories. As mentioned in the pre-test requirements, participants who tapped the table once when they encountered a task event were considered to have successfully completed the target event and were awarded one point. At the end of the card presentation, the participants who volunteered their correct contact information earned two points. The maximum score was 8 points.

Before the TBPM test, participants were instructed to tap on the table every 5 min. The timer was placed 1 m behind the participant’s right shoulder during the test and was used to check the time rather than serve as a reminder. During the 17-min test, after timer activation, the participant was asked to select the largest and smallest numbers on each of the 100 cards presented. When the participant tapped on the table within 10 s before or after the target time, two points were awarded; one point was awarded for tapping on the table within 30 s before or after, and no points were awarded otherwise. The maximum score was 6 points.

### 2.3. Image acquisition

Using a GE Signa 3.0-T scanner, MR images of the brain were obtained using the 3D BRAVO sequence from the AUCM. The main parameters were as follows: repetition time (TR) = 8.16 ms; echo time (TE) = 3.18 ms; flip angle (FA) = 12°; matrix = 256 × 256; field of view (FOV) = 256 × 256 mm^2^; resolution = 1 × 1 mm^2^; slice thickness = 1 mm, with a total of 170 slices scanned. The ears of the participants were plugged with cotton to avoid discomfort from machine noise. Individuals were instructed to remain quiet, close their eyes, and stay completely still during their scan to avoid any degradation of image quality from movement artifacts.

### 2.4. Preprocessing of images

The 3DT1-weighted MR images were acquired in Digital Imaging and Communications in Medicine (DICOM) format at the AUCM. The original DICOM images were converted to NIFTI format using FSL (version 6.0.3). Prior to all steps, images were normalized to the Montreal Neurological Institute (MNI152) standard T1 whole brain template (standard space 91 × 109 × 91, resolution 1 × 1 mm^2^) using FSL. The acquired NIFTI images were imported into FSL, and the hippocampus and basal ganglia mask (91 × 109 × 91) for anatomical automatic labeling was used to set regions of interest (ROIs) for feature extraction.

### 2.5. Radiomic feature extraction

**Figure 1:**
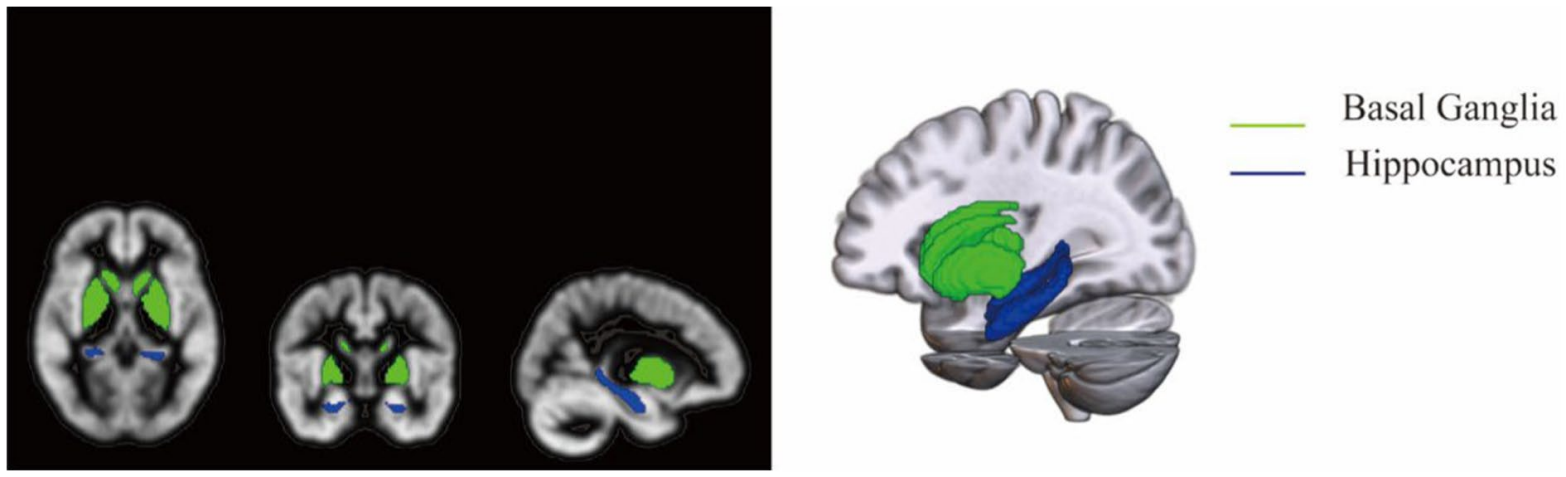
Regions of interest for extracted data.

Radiomic features were extracted from MR images using 3D Slicer (version v4.11.20210226) with the PyRadiomics extension installed. For presentation purposes, 120 radiomic features were extracted from the T1-weighted MR images. The extracted ROIs are shown in Figure 1.

### 2.6. Model building

**Figure 2:**
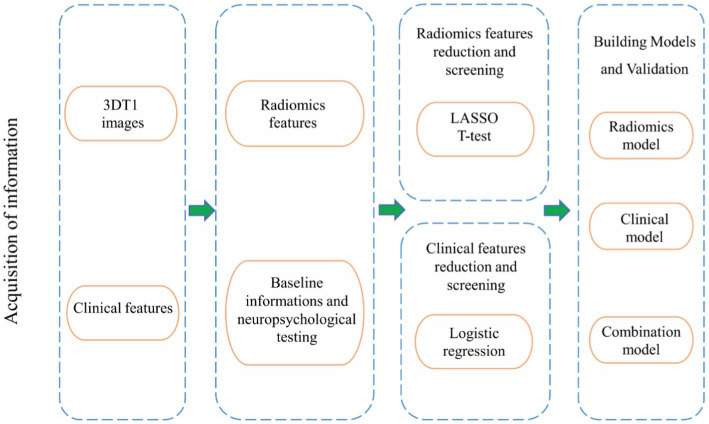
Flow chart of the study design.

The radiomic features obtained for the ROIs were used to build a model for distinguishing between WD and WD cognitive impairment. For comparison, clinical models were constructed using the clinical characteristics of the WD and WD cognitive impairment groups. Thus, an integrated model was constructed through the combination of clinical and radiomic models. The radiomic features were first modeled using a random forest plot, support vector machine, and logistic regression. The clinical characteristics were modeled using logistic regression. The best radiomic and clinical models were then combined to obtain the integrated model. The predictive performance of all models was evaluated using the 10-fold cross-validation method. To avoid sampling errors, 70 and 30% were randomly selected as training and testing datasets. Figure 2 shows the complete modeling process.

### 2.7. Statistical analysis

The implemented models were analyzed based on the area under the receiver operating characteristic (ROC) curve (AUC). Accuracy, sensitivity, and specificity were also evaluated for each model. R (version 4.2.1) was used to generate the ROC curves for each model. The Delong test was used to test the significance of the ROC curves of the different models. *p* values <0.05 were considered statistically significant.

## 3. Results

### 3.1. Baseline data

**Figure 3:**
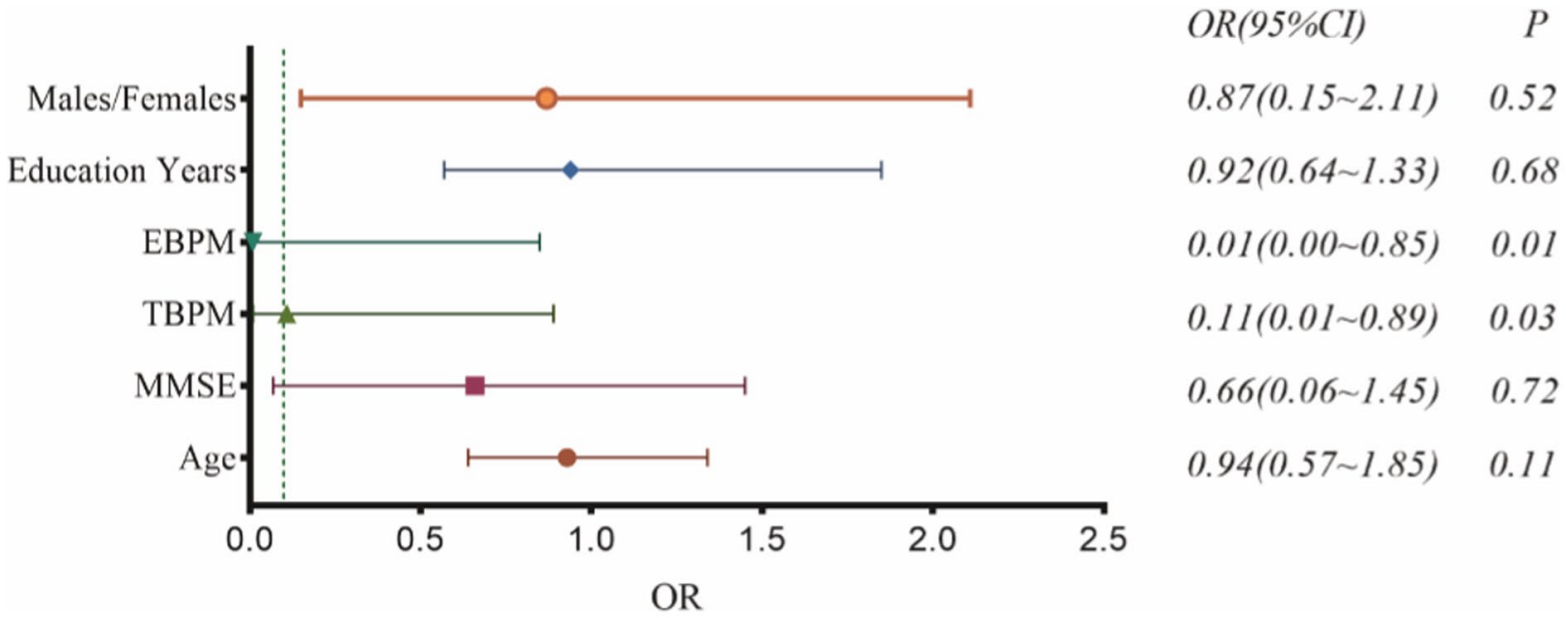
Independent predictors of WD cognitive impairment.

A total of 136 participants underwent MRI and participated in the study, including 77 patients with WD and 59 patients with WD cognitive impairment. No significant differences in the distributions of sex, age, or years of education were observed between the groups (*p* > 0.05); however, MMSE, TBPM, and EBPM scores significantly differed between the WD and WD cognitive impairment groups (*p* < 0.05) (Table 1). Logistic regression analyses revealed that the odds ratios (ORs) for predicting cognitive impairment in WD were 0.87 (0.15–2.11), 0.92 (0.64–1.33), 0.01 (0.00–0.85), 0.11 (0.01–0.89), and 0.66 (0.06–1.45) for sex, years of education, MMSE score, TBPM score, and EBPM score, respectively. TBPM and EBPM scores were identified as independent predictors of WD cognitive impairment (*p* < 0.05) (Figure 3). Based on the results of the logistic regression analysis, clinical prediction models including TBPM and EBPM scores were developed.

**Table 1:** Clinical and biochemical characteristics of patients.

At initial presentation	Patients of WD	Patients of WD with cognitive impairment
Patients (NO.)	77	59
Males/Females (NO.)	38/39	29/30
Ages (years, mean ± SD)	23.81 ± 5.21	24.93 ± 6.67
ACE-III-C	90.45 ± 2.81	81.07 ± 5.42
MMSE^*^	28.19 ± 0.83	27.01 ± 0.83
TBPM^*^	5.06 ± 0.81	3.81 ± 1.47
EBPM^*^	6.55 ± 1.15	4.11 ± 1.01
Education Years (years, mean ± SD)	10.65 ± 1.19	10.70 ± 1.23

^*^Indicates *p* < 0.001, WD versus WD with cognitive impairment in independent *t*-test cohort.

### 3.2. Model building and validation

**Figure 4:**
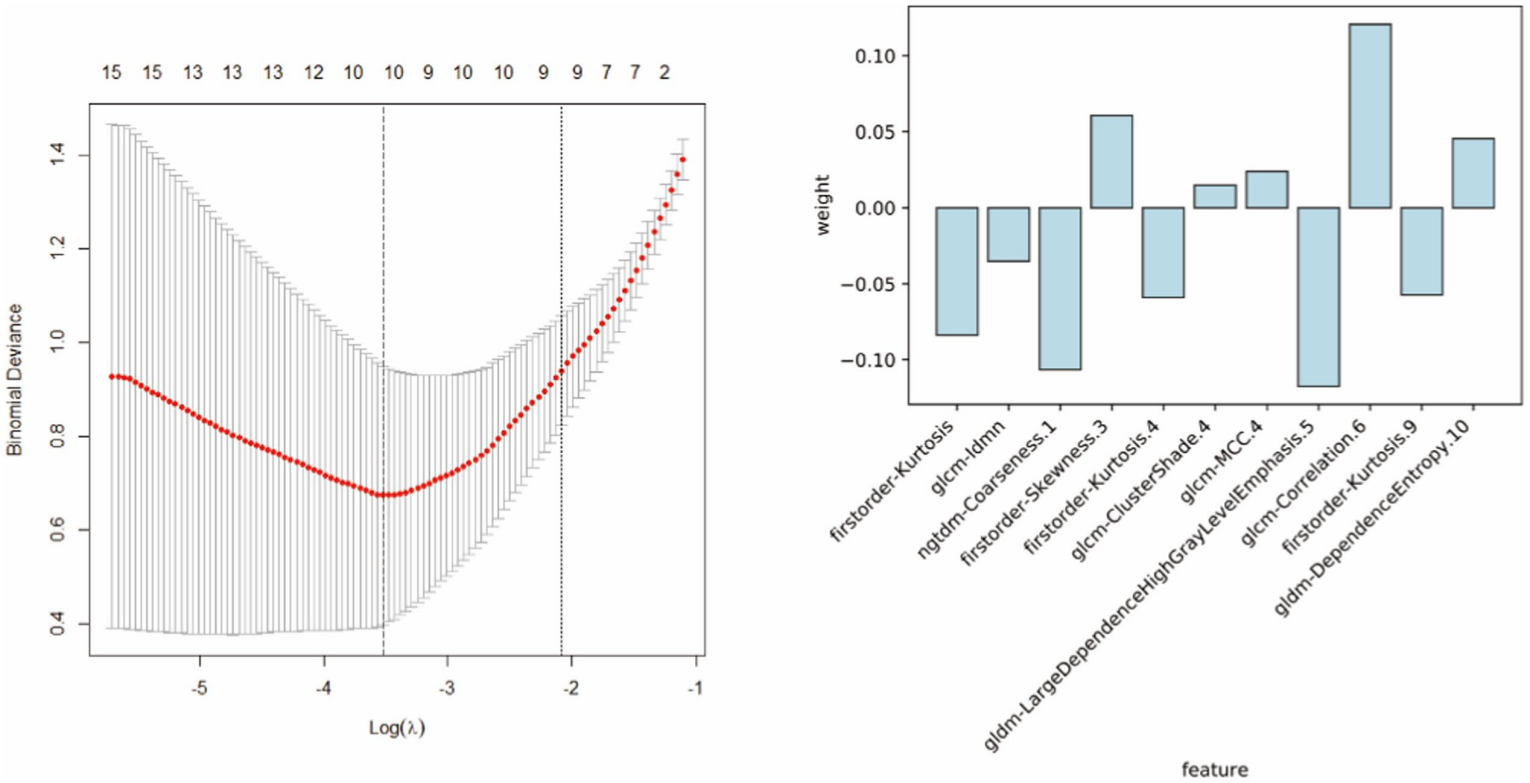
The 11 most optimal radiomic features were screened using *t*-tests and least absolute shrinkage and selection operator (LASSO) regression to construct the radiomic model.

**Figure 5:**
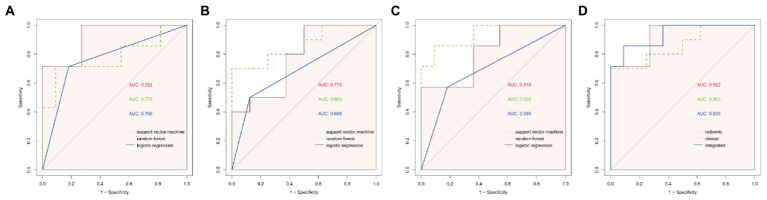
ROC curves of different models. **(A)** The ROC curves of the three modelling approaches of support vector machine (svm), random forest (rf) and logistic regression (logit) for the radiomic model, and the AUC of the svm model was found to be significantly different from that of the logistic regression model by Delong test (*p* = 0.027). **(B)** ROC curves for the three modelling approaches of svm, rf and logit for clinical models, and no statistical significance was found between the three by Delong test (*p* > 0.05). **(C)** ROC curves for the three modelling approaches of ssvm, rf and logit for integrated model, with a significant difference in AUC between the rf model and logit model found by Delong test (*p* = 0.047). **(D)** ROC curves for the best modelling approach for the three models. Combining the results of the AUC and the Delong test, the svm model was chosen for the radiomic model, the rf was chosen for the clinical model and the rf was chosen for the integrated model, and finally the Delong test revealed no statistical significance between the three modelling approaches (*p* > 0.05).

Radiomic features were extracted from patient images, and the 11 most optimal radiomic features were screened using *t*-tests and least absolute shrinkage and selection operator (LASSO) regression to construct the radiomic model (Figure 4). The random forest, support vector machine, and logistic regression models for distinguishing WD and WD cognitive impairment in the test group yielded AUC values of 0.922, 0.779, and 0.766, respectively (Figure 5A). Random forest was found to be the best model for constructing radiomics by the Delong test. Based on the results described in Section “Baseline data” the random forest, support vector machine, and logistic regression models for distinguishing WD and WD cognitive impairment in the test group yielded AUC values of 0.863, 0.775, and 0.688, respectively (Figure 5B). Radiomic features and clinical characteristics were screened together to form integrated model. The random forest, support vector machine, and logistic regression models for distinguishing WD and WD cognitive impairment in the test group yielded AUC values of 0.925, 0.850, and 0.725, respectively (Figure 5C). In the test group, the AUCs for the radiomic, clinical, and integrated models were 0.922, 0.863, and 0.935, respectively (Figure 5D).

**Figure 6:**
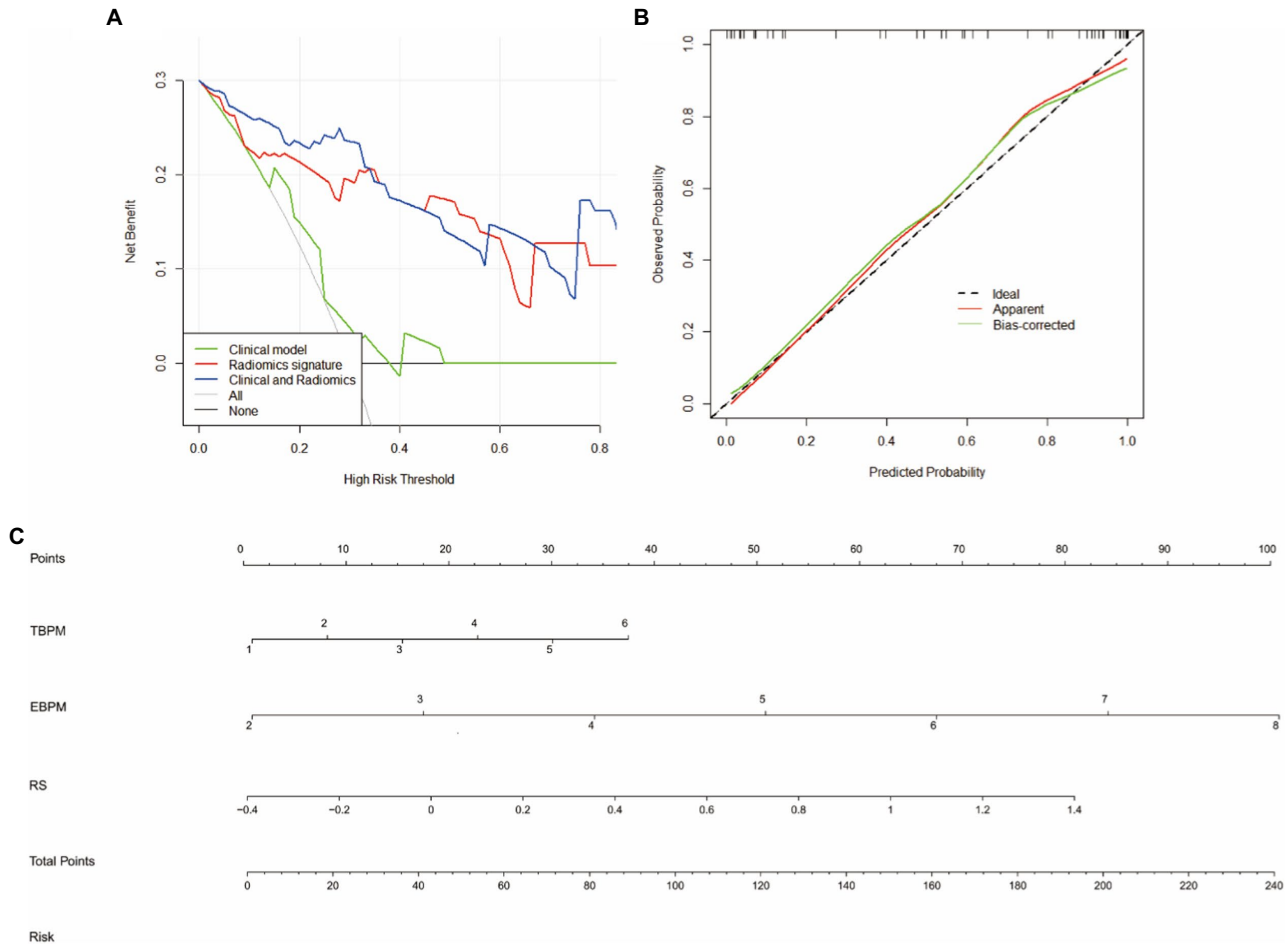
Nomogras, calibration curves, and decision curves for building a model based on radiomic and clinical features. **(A)** Decision curves. Red, green, and blue lines represent the clinical, radiomic, and integrated models, respectively. The *y*-axis indicates the net benefit, while the *x*-axis indicates the threshold probability. The integrated model had a higher overall net gain in distingguishing WD from WD with cognitive impairment when compared with the clinical and radiomic models. **(B)** Calibration curves indicating the goodness of fit of the nomograms. The 45° straight line indicates a perfect match between the actual (*y*-axis) and predicted (*x*-axis) probabilities of the column plots. Closer distances between the two curves indicate higher accuracy. **(C)** Nomograms, time-based prospective memory (TBPM), event-based prospective memory (EBPM), and Radscores were used to build nomograms for clinical use.

The random forest model was identified as the preferred radiomic model and was used to construct a nomogram that combined the Radscore with associated independent predictors of clinical characteristics. In summary, the integrated and radiomic models outperformed the clinical model in the validation groups, and the nomogram of the combination model was considered favorable. The calibration curves revealed good consistency and stability of the model results for predicting the risk of WD cognitive impairment occurrence. The clinical decision curve demonstrated a significant positive effect of the integrated model, indicative of favorable validity (Figure 6).

## 4. Discussion

In the central nervous system, WD mainly damages the neural circuits between the basal ganglia and hippocampus, two regions that are implicated in learning and memory. The hippocampus is extensively connected to the cortex and basal ganglia, playing important roles in cognition, stress responses, and emotion regulation (22). Alterations in hippocampal and basal ganglia circuits are strongly associated with cognitive processing abilities and are currently considered the anatomical basis for the development of common neuropsychiatric disorders (23). Early clinical intervention can delay progression of the disease and improve cognition, highlighting the importance of distinguishing between WD and WD cognitive impairment. Useful interventions include aerobic exercise, medications, and complementary medicine; for example, treatment with penicillamine, zinc salts, and traditional Chinese medicine not only delay copper deposition and the associated liver and kidney damage but also can improve cognitive status in patients with WD (24).

### 4.1. Clinical characteristics of WD and WD cognitive impairment

In medical statistical analysis, which focuses on whether differences exist between variables and outcomes, clinical prediction models have emerged as useful statistical methods. Clinical prediction models aim to combine multiple variables to predict disease outcomes, which are then applied to new datasets to predict disease progression and risk (25, 26). A meta-analysis by Ramanan and colleagues reported varying degrees of PM impairment in patients with neurodegenerative disease, noting that decreases in TBPM scores were more apparent than those in EBPM scores (27). Costa and colleagues argued that both PM and retrospective memory may be impaired in those with mild cognitive impairment. Impairments in declarative memory may be responsible for impairments in retrospective memory, whereas reduced executive ability or deficient reflex mechanisms may explain impaired prospective components (28). Although the diagnosis of WD cognitive impairment is insidious, most patients have irreversible neurological damage including cognitive impairment such that their MMSE scores may be significantly atypical. The above findings are congruent with those of our previous study, in which the majority of patients with WD exhibited cognitive impairment, mainly in TBPM (7). However, further research is required to clarify the value of this finding for the diagnosis and prediction of WD cognitive impairment.

Previous research has identified significant differences in the risk of developing cognitive impairment according to sex (29). Differences in cognitive function among adults may be related to differences observed during neuronal development. Lin et al. reported a much higher risk of death in women than in men in the same group of patients with cognitive impairment, which may have been related to differences in lung function (30). Li et al. proposed that sex-based differences in cognitive function may be correlated with differences in hormone levels (31). Lövdén et al. noted that changes in cognitive ability during human growth and development are associated with a variety of factors, including an increase in time spent in education, which may mitigate age-related decreases in cognitive ability (32). In a systematic evaluation of 71 published papers on the relationship between cognition and education, Sharp et al. reported a correlation between the occurrence of cognitive impairment and educational attainment. There may be some relationship between different levels of education and the risk of developing cognitive impairment; however, this relationship was non-linear and did not reflect the relationship between years of education and cognitive status in equal proportions but was only a rough predictor of cognitive capacity in the broader context of years of education (33, 34). A search of the available literature revealed no published cohort studies on risk factors for the development of WD cognitive impairment, and the mechanisms for the development of WDPMI are mostly described from a neuroimaging perspective. Therefore, large-scale clinical studies are required to provide clinical guidance regarding early detection and intervention in high-risk patients (35, 36).

Sex, years of education, MMSE score, TBPM score, and EBPM score have been commonly identified as predictors of cognitive impairment in patients with neurodegenerative disease (37). Our logistic regression analysis identified TBPM, and EBPM scores as independent predictors of WD cognitive impairment, with EBPM score being the major predictor. The model with clinical features yielded AUC values of 0.863 for the test group, indicative of favorable predictive ability.

### 4.2. Radiomic characteristics of WD and WD cognitive impairment

Conventional MRI may have limited value in some neurodegenerative or psychiatric disorders, such as the inability to visualize the main pathological features of early Alzheimer’s disease (38). Radiomics was first introduced by Lambin et al. in 2012 and has now been successfully applied in research related to oncology, neurodegenerative diseases, and cardiovascular diseases (39–42). Extraction of high-throughput quantitative features for MRI-based radiomics analysis requires a series of sequential steps including image acquisition, image segmentation, feature extraction, and modeling (43). Conventional medical images allow the evaluation of morphological features, textural features, and other characteristics that cannot be captured by visual assessment (39). Therefore, combining radiomic analysis with clinical characteristics can significantly improve the efficiency of diagnosis and classification (44).

In this study, we obtained 11 optimal radiomics features by extracting and filtering MR images, which belonged to the first-order, gray-level co-occurrence matrix, gray-level dependence matrix, and neighboring gray tone difference matrix. First-order refers to first-order feature parameters, while the other second-order parameters reflect the heterogeneity of the roughness and complexity of the image (45, 46). Several previous radiomics studies have demonstrated the beneficial predictive value of radiomics in the differential diagnosis of neurodegenerative diseases (47–49). Studies have also shown that impairments in the basal ganglia can affect learning and memory processes (50, 51). The hippocampus plays a crucial role in memory and cognitive functions (52).

In this study, we established a diagnostic model for WD cognitive impairment based on hippocampal and basal ganglia imaging features, which yielded high diagnostic validity. Additionally, we combined relevant neuropsychological test scores to construct an integrated predictive model and visual nomogram to effectively assess the risk of future cognitive decline in patients with WD. The nomogram based on the integrated radiomic and clinical features may help reveal potential associations between features and disease pathology (52). Nomograms are advantageous when compared to other prediction methods given the inclusion of multiple predictors that are plotted on the same plane using scaled lines to express the interrelationships between the variables in the model, providing individualized risk predictions for each patient (53).

### 4.3. Efficiency of model building

In this study, the AUC values for the validation groups ranged from 0.863–0.935 when the integrated model was applied, yielding a significant diagnostic benefit when compared to traditional models based on radiomic features or clinical characteristics alone. Additionally, both TBPM and EBPM scores were identified as independent predictors of WD cognitive impairment in the multiple logistic regression analysis and were therefore included in the clinical model. Our analysis indicated that, although the clinical and radiomic models could distinguish WD from WD cognitive impairment, the integrated model exhibited better performance. In addition to avoiding overfitting, we validated the nomogram constructed in this study, which is ideal for evaluating model performance (54). The above results suggest that T1-weighted radiomic models of the hippocampus and basal ganglia have high diagnostic efficacy for both WD and WD cognitive impairment. This strategy represents a safe and non-invasive method for monitoring the risk of changes in cognitive status over time. Additionally, models based on radiomic features can provide more comprehensive information about the brain than conventional images, aiding in the development of individualized treatment plans (55).

This study inevitably has some limitations. First, as this was a retrospective study conducted at an individual hospital, selection bias may have occurred, highlighting the need for validation in prospective studies. Second, we utilized a single sequence of MR images, and future studies should employ different sequence types for more in-depth analysis. Furthermore, we used a single MRI machine. Future studies should aim to provide multicenter, multimodal, and standardized clinical data by including independent institutions, different MRI scanning instruments, or databases for external validation. Finally, radiomic analysis has not yet been applied in widespread clinical practice, and these methods must be extensively validated and optimized in further clinical trials. It has to be mentioned that software that specializes in processing cranial MR images, such as FreeSurfer, was not used in this study to extract features including grey matter volume and cortical thickness of each nucleus, etc. It is expected that our next work will improve the shortcomings and regrets of this study. Despite some shortcomings in the analysis of MRI histology, this approach holds great potential as an advanced quantitative method for the diagnosis and prediction of cognitive changes in patients with WD.

## 5. Conclusion

The nomogram developed in the current study—which was based on radiomic features, EBPM results, and TBPM results—is an effective tool for distinguishing WD cognitive impairment from WD and may assist clinicians in the early identification of cognitive impairment in patients with WD. Notably, this quantitative diagnostic modality is non-invasive and reproducible and can help identify the onset of cognitive impairment in WD at an early stage, thus informing treatment planning to improve the long-term prognosis and quality of life of patients.

## Data availability statement

The raw data supporting the conclusions of this article will be made available by the authors, without undue reservation.

## Ethics statement

The studies involving human participants were reviewed and approved by the First Affiliated Hospital of the Anhui University of Chinese Medicine Ethics Committee. Written informed consent to participate in this study was provided by the participants’ legal guardian/next of kin.

## Author contributions

LT: images collection, analyzed the images data, and wrote the manuscript. TD: conceive, supervision, and review. SH, CZ, and HH: investigation and analyzed the data. GY: validation and data collation. WY: supervision and review. All authors contributed to the article and approved the submitted version.

## Funding

This study was supported by the Key Research and Development Program Project of Anhui Province (No.202204295107020043), Natural Science Foundation of Anhui Province (No. 2208085MH270), Natural Science Research Project of Anhui Universities (No. KJ2021A0547), The University Synergy Innovation Program of Anhui Province(NO.GXXT-2020-025) and the National Administration of Traditional Chinese Medicine:2019 Project of building evidence based practice capacity for TCM(No.2019XZZX-NB001).

## Conflict of interest

The authors declare that the research was conducted in the absence of any commercial or financial relationships that could be construed as a potential conflict of interest.

## Publisher’s note

All claims expressed in this article are solely those of the authors and do not necessarily represent those of their affiliated organizations, or those of the publisher, the editors and the reviewers. Any product that may be evaluated in this article, or claim that may be made by its manufacturer, is not guaranteed or endorsed by the publisher.
